# Determinants of client satisfaction to skilled antenatal care services at Southwest of Ethiopia: a cross-sectional facility based survey

**DOI:** 10.1186/s12884-018-2121-6

**Published:** 2018-12-06

**Authors:** Serawit Lakew, Alaso Ankala, Fozia Jemal

**Affiliations:** 1 Department of Nursing and Midwifery, Arbaminch University, Arba Minch, Ethiopia; 2 Department of Nursing and Midwifery, Arba Minch College of Health Sciences, Arba Minch, Ethiopia; 3 Department of Obstetrics and Gynecology, Tikur Anbesa Specialized Hospital, Addis Ababa University, Addis Ababa, Ethiopia

**Keywords:** Satisfaction, Skilled antenatal care, Women, Southwest of Ethiopia

## Abstract

**Background:**

Patient satisfaction to Antenatal care services has traditionally been linked to the quality of services given and the extent to which specific needs are met. Even though data in this area was limited in Ethiopia, improving quality of care was one of the strategies in health sector development program IV. This study, therefore, attempted to assess client satisfaction to skilled antenatal care services in the study area.

**Methods and materials:**

A cross-sectional facility based survey was conducted among women who were attending antenatal care clinic, using quantitative method triangulated with qualitative data collection. Participants were selected using systematic sampling method according to the flow pregnant women to the antenatal care clinics. The study was carried out in all functional public health centers in the district. During the survey, 405 women were interviewed. A logistic regression model was applied to control for confounders.

**Results:**

Out of the total respondents, overall satisfied to skilled antenatal care services were about 277(68%). The most common specific component of antenatal care that had good-satisfaction by the respondents was “Privacy” at examination (81.7%). Most satisfied health education session was “Diet and nutrition” session (82.2%). Absence of sonar test, no doctor and long waiting time were commonest causes of dissatisfaction. Respondents who have > 2 previous antenatal care visit were 3 times more likely (AOR = 2.93; 95% CI, 1.21, 7.12) to have satisfaction to antenatal care services as compared to those with < 1 visit. Women whose current visit fourth were 9 times more likely (AOR = 9.02, 95% CI; 1.76, 46.1) to be satisfied for antenatal services than those who were in the first visit. Women with family monthly income of $US 25–100 per month were 60% (AOR = 0.4, 95% CI; 0.2, 0.8) less likely to have satisfaction by skilled antenatal care services than those who had monthly household income below $US 25.

**Conclusion and recommendation:**

Women who reported good-satisfaction to overall skilled antenatal care services were highest as compared to previous Ethiopian study findings. Demographic, economic, obstetric and distance factors were independent predictors of satisfaction to skilled antenatal care services. Non natives must be encouraged to seek satisfying services.

## Background

Pregnancy is a very important event from both social and medical point of view. ANC is an opportunity to advice the women on how to prepare for complications and promote the benefit of skilled attendance at birth [1].

W.H.O recommends that pregnant women should feel welcome at clinics for ANC in that it should be user-friendly. Examinations and tests should be carried at times that suit the woman. The teamwork between professionals and the pregnant woman is decisive for the safety of the woman and her fetus [2]. Every woman has the right to obtain recommended services of ANC from a skilled attendant at her pregnancy. A skilled attendant is not only trained to attend to normal pregnancies but also to recognize and manage complications and make referrals to hospital if more advanced care is needed. Women in rural areas were most at risk of giving birth and ANC services in the absence of skilled attendant [3, 4].

Ratings of women satisfaction for ANC indicated higher across developing countries and vary from country to country [5–9]. Studies found that overall satisfaction was highest in Cameroon and Egypt. In Cameroon, it was about 96.9% satisfied [10]. In Egypt, more than 90% reported satisfied for waiting time in lab results, Staff help, trust the doctor followed by cleanness of the center, privacy, most of accessibility items, and most of physician performance items. Least satisfied (below 30%) for location of the center, health education program, and explanation of the problems by physicians [11].

In Riyadh, about 87.7% pregnant women attending ANC felt unhappy because they had to wait up to 1 hour before being seen by the physician. About 63.1% were satisfied with information regarding their treatment. Around 18.9% thought that information was not enough and 17.25% reported they did not receive any information about their treatment [12].

In Kenya, about 96% of women who attended FI ANC clinics and 97% who attended NI ANC clinics were either “satisfied” or “very satisfied” with their clinic visit. A ‘very poor’ grade of satisfaction was considered to be a weak area of antenatal services. The dissatisfaction expressed mainly related to the process of imparting health education (such as: commitment, availability of time and language barrier) and not to the availability of health education material [13].

In Nigeria, most respondents were satisfied by the services given at the clinic (81.1%). Sitting arrangement was most satisfied (97.9%). Toilet and bathroom facilities were least satisfied (39.3 and 38.1% respectively). About 91.6% respondents reported “diet and nutrition” more satisfied during the interactive session than others. Prevention of cervical cancer was least discussed topic (65.7%) [14]. Obafemi Awolowo University study findings added that about 55% of women attending ANC clinic were satisfied with the quality of health talk. Around 72.6% were satisfied by the opinion that the services of the hospital were good and met their needs. About 53.7% agreed with the competency of the hospital staff. About 39.1% agreed with timely response of the staff and 20.5% on the opinion that the staffs were friendly and polite [15].

In various African country, Continued utilization of ANC services in the future pregnancy was directly linked to the satisfaction of the clients (*p* < 0.05) [14]. Significant satisfaction was also observed for being attending in public centers over private in Cameroon and those who served in Mendera Kochi and Higher Two Centers over others at Jimma town of Ethiopia [10, 16]. Those who have no formal education, attended primary education, monthly income < 500 birr and between 750 and 1000 birr, planned pregnancy and no history of stillbirth had significant associations for satisfied ANC services over their counter parts at *p* < 0.05 [16].

In Cameroon, significant association was observed for good satisfaction at their first pregnancy, the sitting area comfort, and competence of staff as compared to its counterparts [10]. Pakistan qualitative discussants reported, distant location of facilities, lack of functional equipment, medicines and supplies as perceived poor satisfaction [17]. Previous Ethiopian studies focused on the relationship between women’s satisfaction and the provision and utilization of health care services [18]. No data could have been found on specific component of services satisfaction in Ethiopia in general and the study area in particular. This study, therefore, attempted to place its contribution through its findings.

### Conceptual frame work

**Fig. 1:**
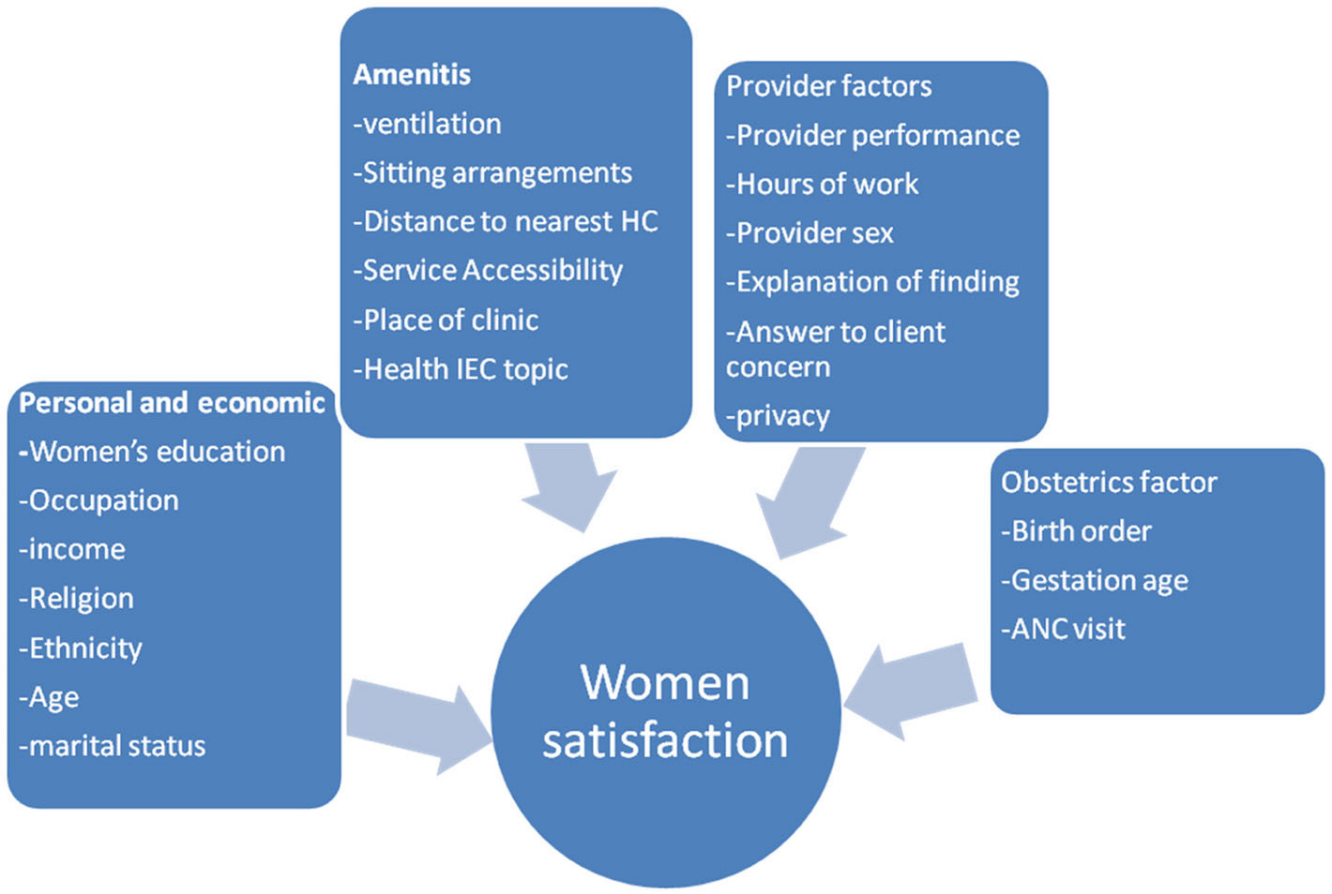
Conceptual Framework showing predictor and outcome variables of the study

As indicated by Fig. 1, this conceptual frame work was adopted from one systematic review on women satisfaction to maternity care services in developing world, since it matches with this local system [9]. ANC services satisfaction was one component with background characteristics, provider factors, obstetrics factors and amenities were independent predictors. The frame work was adjusted to local system for to make it more convenient (Fig. 1).

## Methods

### Study design and setting

Health facility based cross-sectional study was conducted from Jan 1 to March 12, 2016. Health Facility includes Hospitals, Health Centers, Health Posts and Private Clinics. Arba Minch Zuria was one of the districts in the southwest of Ethiopia. It was located in the Great Rift Valley. As per the report of district health department statistics office, it had 30 kebeles (kebele is the lowest administrative state in Ethiopia) and a total population of 202,495 of whom 100,842 are men and 101,653 women. The district was located some 454kms southwest of Addis Ababa. The district had no any functional hospitals. But, it had five functional health centers and about thirty health posts (staffed by Health Extension Workers who are non-skilled providers) and private clinics together [19, 20]. The private clinics in the study region had no routine ANC services, since it had service fee and costly. Routine ANC services exist only in government health centers because services were free. In the district, skilled providers exist in the public health centers and private clinics only. Health Centers, therefore, were selected as study unit.

### Sample size and sampling procedure

**Fig. 2:**
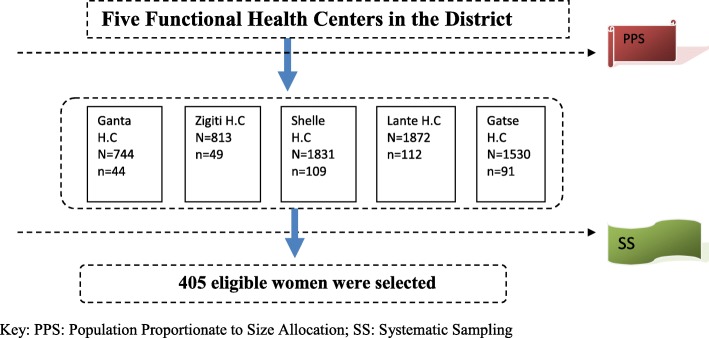
Schematic presentation of sampling procedure and selection

Sample size was determined by using single population proportion (SPP) formula based on the assumptions of 95% confidence level, 60.4% *p*-value (previous Rural Ethiopian study) and a 10% contingency. Accordingly, the total sample size was 405 women participant. Systematic sampling technique was used to select the study subjects. Number of Participants in each health centers was estimated based on three steps. First, total catchment population to each health centers were obtained from district health offices, department of statistics. Next, estimated proportion (N) of pregnancy in the catchment population of each health centers (using 4.5% of the whole population [21]) was obtained. Finally, the number of attendance for ANC (using 34% [22]) within the total estimated pregnancy was calculated in each health center. Respondents (n) who were registered for antenatal care during data collection at post-procedure period were interviewed by systematic sampling technique until the required sample size was achieved in each health center. The K^th^ value was calculated based on number of registered for ANC of the day divided by expected sample a day (in each health center). From the first K^th^ values, one woman was selected by lottery method. The consecutive woman was selected by every k^th^ value. Also consider that ANC registration was performing in the morning for rural Ethiopian Health facility as tradition (Fig. 2).

For qualitative method, a convenience sampling technique was used to select pregnant women participant for the FGD by taking health center as homogeneity criteria. Accordingly, 10 FGDs were purposively selected with 2 FGDs in each of five active health centers. Selected respondents were not participant on quantitative. FGDs were conducted on two occasions with different days after the end of quantitative interview. It was accomplished by same data collectors under supervision.

### Questionnaire development and measurement

Questionnaire was adapted from previous similar studies in the abroad [11] and adjusted in the local system. The questions and statements were grouped and arranged according to the particular that they can address. After extensive revision, the final version of the English questionnaire was developed. An individual who were expert for English and Amharic languages translated the English version into Amharic and the vice versa. For quantitative method: data were collected using face-to-face client interview questionnaires (by exit interview). Having skilled Antenatal Care services were those participant who had ANC by skilled Health personnel, such as Nurse, Midwife, Health Officer and/or Doctor at Health Facility. Having good satisfaction to Skilled Antenatal Care services were those women who answered ‘good’ to satisfaction questions in general and the vice versa for poor satisfaction. Predictor variables included in the data collection tool were demographic and Socio-economic variables; obstetrics variables; provider and staff variables; and amenities. For qualitative method: Focus group interview guideline was used to guide and probe the FGDs. The FGD interview guideline includes probing questions on areas of care clients satisfied and areas of care clients not satisfied. About 6–12 volunteered discussants were selected for each FGD session from each health centers. Every participant had given chance to talk as per the need to talk.

### Statistical analysis

For quantitative method, after data collection each questionnaire was manually checked for completeness and then coded. After this validation, data were entered using EPI INFO version 3.5.2 and exported to SPSS statistical software package windows version 20 for analysis. Descriptive and summary statistics were used to describe the study population by variables of interest based on conceptual framework and for outliers. The degree of association between independent and dependent variables were described using crude and adjusted odds ratio. Bivariate analysis was applied to examine the association between each of the independent variables and satisfaction of ANC services. Multivariate statistical method used in the analysis was logistic regression model to control confounders. For qualitative methods, FGD data transcribed into English language verbatim, read critically and essential themes were identified. Ideas related to various themes were color coded and then organized into concepts and presented using narratives. The result was presented in tri-angulations with the quantitative data using subjects verbatim as illustrations.

### Data quality control

Data Collection tool was adopted and pretested [11]. Three days Training was given to data collectors and supervisors. Every day, completed questionnaires were reviewed and checked for completeness and relevance by the supervisors. All the necessary feedback was offered to data collectors in the next morning before the actual procedure. Data checked for completeness, coded, entered into computer, cleaned, and frequency checked for outliers and missing values before analysis.

### Ethical issues

Ethical clearance was obtained from Arba Minch College of Health Sciences Research and Publication core process (RPCP) and Southern National regional state Health Bureau. The study was commenced after letter of cooperation written to each catchment health facility administrators from Zonal administrators (ZHB). Informed written consent was secured to all study subjects. Each respondent was informed for the objective of the study and assurance of confidentiality, risks and benefits. List of Colleges Ethics committee includes: Alemayehu Bekele Kassahun, Addisu Alemayehu Gube, Tarekegn Tadesse Hunede, and Bereket Workalemahu Ayele.

## Results

### Socio-demographic and personal characteristics

Of the total 405 sample, the response rate was 100%. Of the age distribution of the women, about 315 (77.8%) of the participant were the dominant group of 20–34 years. The mean age of the participant was 27.6 years ± 5.6 SD. Concerning woman education, more than half 203 (50.1%) had reported no history of formal education. Gamo ethnic group were the dominant 346 (85.4%) over others among the attendee participated. Except the few, most of women arrived the health facility traveling > 1 km from their home, around 323 (79.8%) (Table 1).

**Table 1:** Independent variables used in the analysis categories and percentage distribution, Arba Minch Zuria district, Southwest Ethiopia, March 2016

Variable	Status of Satisfaction, (*n* = 405)	
	Good satisfaction, n (%)	Poor satisfaction, n (%)
Age		
< 20 yrs	26(6.4)	16(4.0)
20-34 yrs	212(52.3)	103(25.4)
> 35 yrs	39(9.6)	9(2.2)
Woman education		
No education	153(37.8)	50(12.3)
primary	81(20.0)	56(13.8)
Secondary+	43(10.6)	22(5.4)
Distance to the nearest HC		
< 1 km	42(10.4)	40(9.9)
> 1 km	235(58.0)	88(21.7)
Ethnicity		
Gamo	255(63.0)	91(22.5)
Welayta	16(4.0)	24(5.9)
Others^a^	6(1.5)	13(3.2)
Birth order (*n* = 318)^d^		
1	48(15.1)	32(10.1)
2–3	106(33.3)	47(14.8)
4–5	49(15.4)	14(4.4)
6+	19(6.0)	3(0.9)
Previous ANC visit		
< 2 visit	195(48.1)	110(27.2)
> 2 visit	82(20.2)	18(4.4)
Marital Status		
Married	274(67.7)	118(29.1)
Others^b^	3(0.7)	10(2.5)
Religion		
Orthodox	123(30.4)	67(16.5)
Protestant	129(31.9)	58(14.3)
Others^c^	25(6.2)	3(0.7)
Family Monthly Income (*n* = 360)^d^ (in $US)		
< 25	85(23.6)	23(6.4)
25–50	105(29.2)	65(18.1)
> 50	53(14.7)	29(8.1)

N.B: ^a^ include Zayse and Amhara, ^b^ widowed, divorced & single, ^c^ Muslim, catholic, traditional, ^d^ had missing values

Regarding respondents religion, Ethiopian Orthodox and Protestant Christianity religion followers alone account for 377 (93.1%) of the respondents. This showed the two religion dominance in the study region. Birth order of majority of woman was between 2 to 5 among the study participants, about 67.9%. When observing income, net monthly income of the women as family income grouping was higher in middle income categories ($US 25–50), accounting 218 (53.8%) of respondents. The median monthly income of the family was $US 37.5 (Table 1).

### Satisfaction by components of skilled ANC services

**Fig. 3:**
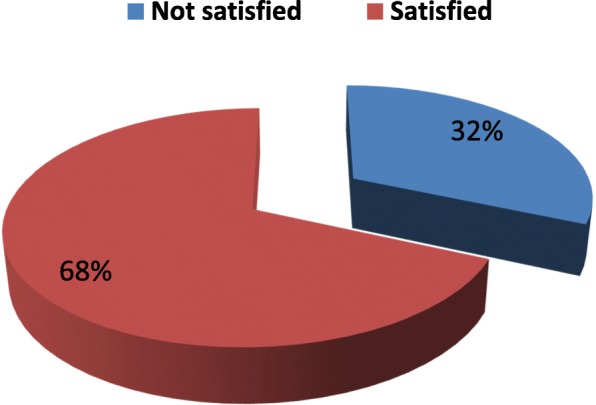
Percent description of overall client satisfaction to the Skilled ANC services in Arba Mich Zuria district, Southwest of Ethiopia, February 2016

Overall satisfaction was classified as poor or good satisfaction. Most women were satisfied with the services offered, about 277(68%). Satisfaction among components of services range from 24 to 82% satisfied. More importantly, satisfaction status was specifically described based on main components of services for Skilled Antenatal Care. Regarding accessibility for services, more women satisfied for staff hours of work 247 (61%) and sitting arrangements 289 (71.4%). Half and more of the women were satisfied by cleanness of the Center 241 (59.5%) and Toilet facility 226 (55.8%) (Table 2 and Fig. 3).

**Table 2:** Percent distribution of level of women Satisfaction to different components of ANC services, Arba Minch Zuria district, Southwest Ethiopia, March 2016 (*n* = 405)

Aspects of Care	Status of Satisfaction			
	Good Satisfied		Poor satisfied	
	Number	%	Number	%
Overall satisfaction to ANC services	277	68.4	128	31.6
Amenities				
Location of the center	194	47.9%	211	52.1%
Hours of work	247	61%	158	39%
Ventilation	196	48.4%	219	51.6%
Sitting arrangement	289	71.4%	116	28.6%
Cleanness of the center	241	59.5%	164	40.5%
Cleanness of the Toilet	226	55.8%	179	44.2%
Performance of provider				
Answering questions	290	71.6%	115	28.4%
Taking history	255	63%	150	37%
Explanation of problem	271	66.9%	134	33.1%
Trusting the doctor	289	71.4%	116	28.7%
Examination time	309	76.3%	96	23.7%
Explanation of rational for investigation	277	68.4%	128	31.6%
Explanation of results of Investigation	241	59.5%	164	40.5%
Performance of staff				
Delivering of information	269	66.4%	136	33.6%
Maintaining Privacy	331	81.7%	74	18.3%

Provider “examination time” client satisfied was far large 309 (76.3%) among others within “performance of provider” responses category as women report suggested and relatively lower responses observed in “explanation of results of investigation”, about 241 (59.5%). Major ‘poor satisfaction’ response in this category was ‘location of the center’ among others, about 211 (52.1%). Regarding privacy, poor-satisfaction report was 74 (18.3%), which is the lowest of all and the vice versa was the highest (Table 2).

Only about a third of the women had good satisfaction to explanation of the problem, explanation of rationale of the investigation on provider performance component, and delivering of the information in staff performance component, about (33.1, 31.6, and 33.6%), respectively (Table 2).

Majority of FGD respondents also reported that most providers had no respect for working hour’s punctuality. For that reason our waiting time was beyond expected time to wait. Most of wasting time occurs on either exit time end or entrance beginning. Once they start the procedure, time utilization was effective. One 21 years old lady said, now I decided to visit the facility not based on government working hours, but providers working hours.

### Describing satisfaction by health education and communication session in the ANC clinic

The health education and communication session on women satisfaction during ANC visit observed that overall health education program was not-satisfied by the majorities, about 245 (60.5%) of the respondents. When specifically observing, cervical cancer prevention IEC session was not satisfied by nearly half of women, about 206 (50.9%). The ‘no-satisfaction’ respondents report were highest for STI prevention, about 268 (66.1%), malaria prevention 214 (52.9%), Physical exercise 212 (52.4%), and Breast self examinations 302 (74.6%) (Table 3).

**Table 3:** Percent distribution of women satisfaction to health education and communication session on ANC clinic, Arba Minch Zuria district, Southwest Ethiopia, March 2016 (*n* = 405)

Health Education Topics	Status of Satisfaction			
	Good Satisfied		Poor satisfied	
	Number	%	Number	%
Overall Health education program	160	39.5%	245	60.5%
Prevention of Cervical Cancer	96	23.7%	309	76.3%
STI’s prevention	137	33.8%	268	66.1%
Malaria prevention	191	47.2%	214	52.9%
Physical exercise	193	47.7%	212	52.4%
Personal hygiene	284	70.1%	121	29.8%
Teeth care	214	52.8%	191	47.2%
Diet and nutrition	333	82.2%	72	17.8%
Clothing	277	68.4%	128	31.6%
Fetal movement monitoring	256	63.2%	149	36.8%
Allowable medication	276	68.1%	129	31.9%
Breast feeding importance	247	61%	158	39%
Basics of newborn care	211	52.1%	194	47.9%
Follow-up appointment	303	74.8%	102	25.2%
Breast Self examination	103	25.4%	302	74.6%
Signs of labor	288	71.1%	117	28.8%
Danger signs of pregnancy	286	70.6%	119	29.4%
Family planning and child spacing	300	74.1%	105	25.9%

Highest client ‘Good satisfaction’ reports were observed as compared to “poor satisfaction” on some specific sessions. These included personal hygiene 284 (70.1%), tooth care 214 (52.8%), diet and nutrition 333 (82.2%), clothing 277 (68.4%), fetal movement monitoring 256 (63.2%), allowable medication 276 (68.1%), importance of breast feeding 247 (61%), basics of newborn care 211 (52.1%), follow-up for appointment 303 (74.8%), signs of labor 288 (71.1%), danger signs 286 (70.6%), and family planning and child spacing sessions 300 (74.1%) (Table 3). Majority discussants of focus group reported the major Health Education areas focused by the providers were diet at pregnancy and personal hygiene and its importance. We were happy with the sessions given. We identified healthier dietary selection for a pregnant woman. Providers also encouraged us to increase daily consumption of meal in that we have to have at least an additional two meal plan per a day over the routine. A single 34 years old respondent added “a provider told me to continue with the food that I was using even before pregnancy. Such as: vegetable diets, cereals and legumes that I get it from my farm land. Provider also addressed to balance my diet plan with my current weight and weight gain throughout the progress of pregnancy”.

### Describing satisfaction by number of current visit and gestational age

**Fig. 4:**
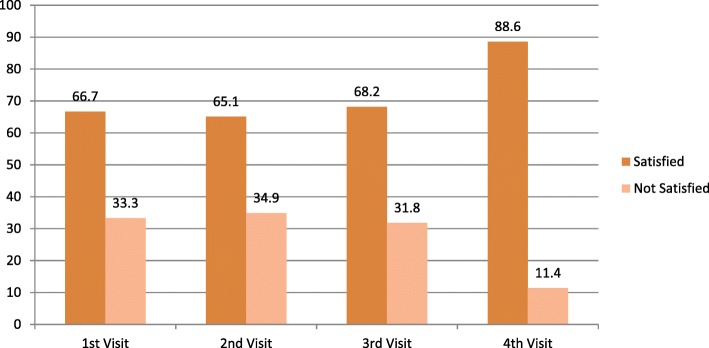
Percent description of women current ANC visit by satisfaction status to ANC services, Arba Minch Zuria district, Southwest Ethiopia, March 2016 (*n* = 405)

**Fig. 5:**
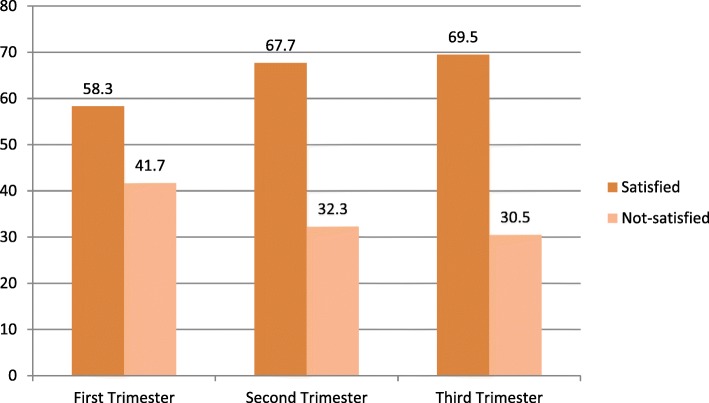
Percent description of women gestational age of current pregnancy visit by satisfaction status to ANC services, Arba Minch Zuria district, Southwest Ethiopia, March 2016 (*n* = 405)

As observed, ‘women satisfied’ was increasing with frequently visiting for this pregnancy. Women satisfied were observed among the fourth and more visits of the respondents (88.6% Vs 66.7, 65.1, and 68.2%). Regarding gestational age, similar phenomena were observed in that in all trimester most women reported “good satisfaction” over that of “poor satisfaction”. But, first trimester satisfaction and dissatisfaction was nearly closer each other (58.3 and 41.7% Vs 67.7 and 32.3%; 69.5 and 30.5%) as compared to second and third trimester. Third and second trimester satisfaction was huge over first, about (69.5 and 67.7% Vs 58.3%), respectively. The average gestational age at first visit of this pregnancy visit was 21.1 weeks ± 7.2 SD (Figs. 4 and 5).

### Perceived main causes of dissatisfaction to skilled ANC services

**Fig. 6:**
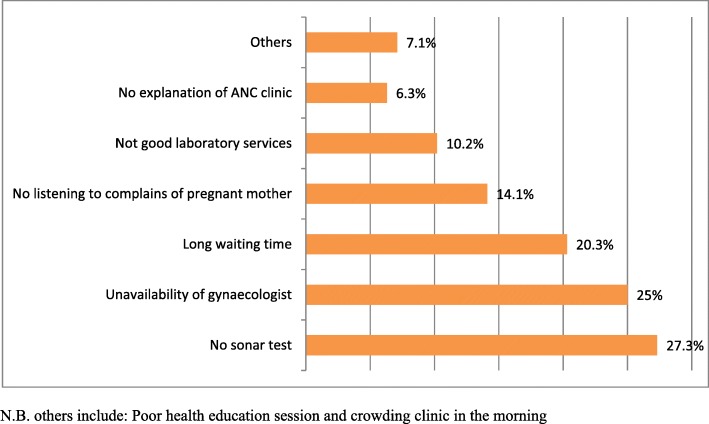
Percent distribution of women by main causes of dissatisfaction to skilled ANC services, Arba Minch Zuria district, Southwest Ethiopia, March 2016 (*n* = 128)

A total of about 128 (32%) respondents had reported for having no satisfaction to Skilled Antenatal Care services in the facility of study district. Among this, reports of no sonar test (27.3%), unavailability of gynecologists (25%) and long waiting time in the clinic (20.3%) took the largest part. Even though they were lower ‘no good laboratory services’, ‘not explaining about the clinic’, ‘overcrowding’, and ‘poor education session’ reports also took proportions as the causes of dissatisfaction for overall Skilled Antenatal Care services (Fig. 6). In addition to this some of the discussants forwarded ‘up on shortage of some medical instruments, providers usually used to refer us to the distant located hospital. Because of this, most of women did not go because of transportation and accommodation cost problem that we encounter at urban hospital and also hospital service was inconvenient than that of rural Health Centers’. A single 28 years old woman among the discussants of Gatse Health Center said ‘I was not happy on the centers timely examination. There is long waiting and now I waited for long time’.

### Factors associated with satisfaction to skilled ANC services

Bivariate and multivariable logistic regression analysis was used to calculate odds ratios and corresponding 95% confidence intervals for the predictors of satisfaction to Skilled Antenatal Care services. Concerning predictors of satisfaction in the bivariate analysis, having satisfaction to Skilled Antenatal Care services was associated with having birth order, distance to the nearest health center, previous ANC visit, family monthly income, marital status, ethnicity, and current visit type (Table 4).

**Table 4:** Adjusted and unadjusted odds ratio of logistic regression model showing effects of predictor variables on the likely hood of client satisfaction for skilled ANC services, Arba Minch Zuria District, South West Ethiopia, March 2016

Predictor variables		Had satisfaction							
		Yes *n* = 277, 68.4%	No *n* = 128, 31.6%	COR	95% CI		AOR	95% CI	
					lower	upper		lower	upper
Birth Order (*n* = 318)	1	15.1%	10.1%	1.0+			1.0+		
	2–3	33.3%	14.8%	1.5	0.85	2.64	1.14	0.58	2.26
	4–5	15.4%	4.4%	2.33*	1.11	4.91	1.55	0.66	3.63
	6+	6%	0.9%	4.22*	1.15	15.4	3.45	0.38	31.4
Distance to the nearest HC^a^	< 1 km	9.9%	10.4%	1.0+			1.0+		
	> 1 km	21.7%	58%	2.54*	1.55	4.18	2.27**	1.27	4.06
Previous ANC visit	< 1	48.1%	27.2%	1.0+			1.0+		
	> 2	4.4%	20.2%	2.57*	1.47	4.5	2.93**	1.21	7.12
Family monthly income ($US)	< 25	23.6%	6.4%	1.0+			1.0+		
	25–50	29.2%	18.1%	0.44*	0.25	0.76	0.4**	0.2	0.8
	> 50	14.7%	8.1%	0.49*	0.26	0.94	0.67	0.27	1.67
Marital Status	Married	67.7%	29.1%	7.74*	2.09	28.6	8.17**	1.43	46.5
	Others^b^	0.7%	2.5%	1.0+			1.0+		
Ethnicity	Gamo	63%	22.5%	1.0+			1.0+		
	Welayta	4%	5.9%	0.24*	0.12	0.47	0.26**	0.13	0.54
	Others^c^	1.5%	3.2%	0.16*	0.06	0.45	0.2**	0.07	0.61
Current Visit	First	25.2%	12.6%	1.0+			1.0+		
	Second	20.7%	11.1%	0.93	0.57	1.53	1.09	0.57	2.08
	Third	14.8%	6.%	1.07	0.61	1.88	1.23	0.55	2.73
	Fourth and more	7.7%	1%	3.87*	1.3	11.57	9.02**	1.76	46.1

N.B: **Statistically Significant Association (*p* < 0.05), *significant by binary analysis, ^1.0+^ Reference category, ^a^Health Center, ^b^Singe, divorced, widowed, ^c^Zeyse, Amhara

When adjusted for other confounders in multivariable logistic regression analyses, only six variables, distance to the nearest health center, previous ANC visit, family monthly income, marital status, ethnicity, and current visit type were the potent predictors of women satisfaction to Skilled Antenatal Care services. Woman who arrived from 1 km or more distance to the center were 2.27 times more likely (AOR = 2.27; 95% CI, 1.27, 4.06) to have satisfied to Skilled Antenatal Care than those who are from below 1 km distance (Table 4).

Respondents who had 2 or more previous ANC visits were three times (AOR = 2.93; 95% CI, 1.21, 7.12) more likely to be satisfied to ANC services as compared to those with less or equal to one visit. Those women with family monthly income of $US 25–100 per month were 60% (AOR = 0.4, 95% CI; 0.2, 0.8) less likely to be satisfied by Skilled Antenatal Care services than those who had monthly household income below $US 25 (Table 4).

Compared to unmarried/divorced/widowed, married women were 8.17 times (AOR = 8.17, 95% CI; 1.43, 46.5) more likely to be satisfied to Skilled Antenatal Care. Non-native (Amhara, Zayse) ethnic groups were 80% (AOR = 0.2, 95% CI; 0.07, 0.61) decreased to have satisfied as compared to the native (Gamo) ethnic groups. And, women who come for fourth ANC visit were 9 times (AOR = 9.02, 95% CI; 1.76, 46.1) more likely to be satisfied than those who were in the first visit (Table 4).

## Discussion

This health institution based cross-sectional study was attempted to assess the satisfaction status of women to Skilled Antenatal Care services in the district of Arba Minch Zuria, Southwest of Ethiopia. Findings of this study showed Skilled Antenatal Care services satisfaction were associated with demographic, economic, obstetric and accessibility factors of the respondents.

Accordingly, high number of respondents had reported they had good-satisfaction to the services. Many studies suggested that satisfaction of clients to ANC services varied from country to country [5–8]. This was inconsistent and higher finding as compared to Oman and previous Ethiopian (68% Vs 59% & 60%) [13, 16] and lower than Nigerian, Kenyan and Cameroons (68% Vs 81.1, 95, 96.4%) [10, 14, 23]. The highest finding could be an indication in Ethiopia for the ever-growing health sector development program for quality services so that it could be contributed to this comparative greater satisfaction. Conversely, the lower finding could be related to still the existence of comparative quality shortages in the study area of Ethiopia.

Satisfaction regarding location of the center, health education program and provider explanation of the problems on women ranges from about one-third to two-third, such as 39.5 to 66.9%. This was higher than Peruvian, Pakistan, European, and two African findings (39.5 to 66.9% Vs < 30%) [5–8, 17]. This higher finding of Ethiopia could be related to minimum surface area of the center due to few departments and buildings. This could give the center location closer to the roadside. Ethiopian provider usually had few minutes session for health education and explanation of the problem to the client. Greater satisfaction in this regard could be due to this short duration in the absence of intervention or treatment process that satisfies most of busy Ethiopian women to go to their home for home based work.

This study showed more than half women had satisfaction by ‘hours of work’ (61%). It was lower finding as compared to African Egyptian study finding (61% Vs 77.9%) [11]. This difference could be due to minimal working hours passed on work by providers in the facility of Ethiopia as per 24 h a day and 7 days a week. Shortage of skilled provider was also another issue to be considered in Ethiopia. Existence of these two facts alone were reported by Ethiopian HSDP-IV of 2012 report [24]. This comparative lower finding was also strengthened by majority of discussants that “………every health professionals within the department on which they were in-charge had not found at government work hours that as we know, we mean at early 2:30 to 3:00hrs morning and 8:00 hrs to 8:30 hrs afternoon. Some exist in the facility compound, but not availed in the department on time. Some others totally not availed, but come late. Others, but not all, exit the facility early, before exit time (exit time: 6:30 morning or 11:30 after noon). It is most disappointing to all of us………….thanks to opportunity to talk”.

In this study, good satisfaction to sitting arrangements, ventilation and cleanness of the toilet reported were nearly ¾^th^ (71.4%), below half (48.4%), and more than half (55.8%) of the respondents, respectively. These were still lower from the highest findings of Nigeria (71.4, 48.4, 59.5% Vs 97.5, 84.1, 60.7%) [14]. This could show still insufficient arrangements for the three variables here in Ethiopia in the extent of its proper functioning to until it brings highest good satisfaction as compared to Nigeria’s center with-in the continent of Africa.

Regarding performance of the provider and staff, all the findings of ‘good satisfaction’ in the current study were more than average as reported, such as: answering questions (71.6%), taking history (63%), trusting the provider (71.4%), examination time (76.3%), explanation of rational for investigation (68.4%), explanation of results of Investigations (59.5%) delivering information (66.4%) and maintaining privacy (81.7%). All of the mentioned components of services were comparatively lower from the Shawa Village Egyptian finding [11]. The probable explanation in this regard could be shortage of professional ethics, availability of non-women friendly services, and performance negligence’s by professionals were dominating here in Ethiopian as compared to outside higher satisfaction countries. As it is mentioned in various studies (such as: Bangladesh and India) [25–27], high satisfaction was also related to Maintenance of privacy via a separate room or screen for examination.

This study assessed ‘poor- satisfaction’ health education and communication sessions as components of services. These included: Prevention of Cervical cancer, STI’s prevention, Malaria prevention, Physical exercise, and Breast Self examination. On the other hand, more than average women reported for each of major HE sessions as ‘good-satisfaction’ included: Personal hygiene, Teeth care, Diet and nutrition, Clothing, Fetal movement monitoring, Allowable medications, Breast feeding, Basics of newborn care, Follow-up appointment, Signs of labor, Danger signs of pregnancy, and Family planning and child spacing. The poor satisfaction was contrary to Nigerian finding [14]. This could be due to not addressing these specific tasks on HE session here in Ethiopia by majorities of providers as contrasted by Nigeria. The good satisfaction components was in-line with Nigerian and Cameroon findings [10, 14]. The positive relationship could be due to increasing health sector development program for education session development here and there.

More than average women reported for each of major HE sessions as ‘good-satisfaction’ included: Personal hygiene, Teeth care, Diet and nutrition, Clothing, Fetal movement monitoring, Allowable medications, Breast feeding, Basics of newborn care, Follow-up appointment, Signs of labor, Danger signs of pregnancy, and family planning and child spacing. This was in-line with Nigerian and Cameroon findings [10, 14]. This positive relation could be due to increasing health sector development program for education session development here and there. Focus group discussants also supported positive finding in that “…….ohhh, it was good and interesting. Health education session focusing on personal hygiene, diet and nutrition, and communicable disease prevention were clear to all of us. After education we have got special things to ourselves and our life. Our child can be protected from future infections, especially diarrhea and vomiting problems. One 38yrs old woman said: Now I will breast feed only for six months exclusively. Vaccinating baby is beneficial to me and to him”.

Causes of dissatisfaction as client reported in the facility were: absence of sonar test, no doctor and long waiting time in the clinic. Other studies (Ghana, Nigeria and Ethiopia) also supported this as causes of dissatisfaction with services [16, 28, 29]. But, in this study, sonar test was the pioneer that reported by the majority of respondents as compared to other findings.

This study observed that having satisfied to Skilled Antenatal Care services was statistically significantly associated (AOR = 2.27; 95% CI, 1.27, 4.06) with distance > 1 km that women traveled to arrive to the nearest Health Center. This was inconsistent with Pakistan qualitative finding [17] as far distance was one of the factors for dissatisfaction. This difference could be due to reduced expectations of women from outside village that she could get adequate services of her need. As suggested by developing countries study, women with low expectations with more services could result in good satisfaction of services and the vice versa [9].

Women Satisfied to SANC services was significantly associated (AOR = 2.93; 95% CI, 1.21, 7.12) with frequent (> 1times) previous ANC visits that women had. This was directly linked to Ibadan, Nigerian finding [14] and Riyadh [12] in which patient satisfaction was significantly higher among women in the highest visit groups. The positive association in this regard could be due to developing awareness on its importance by repeated visiting, increasing client need and effective response to this need by the health care workers here and there. Moreover, satisfied woman are more likely to increase the compliance with ANC visits [30]. Therefore, majority of subsequently visiting women could probably be satisfied groups in the current study and others.

In this study, having satisfied to Skilled Antenatal Care services was significantly associated (AOR = 0.4, 95% CI; 0.2, 0.8) with respondents who had monthly family income of $U.S 25–50/month. This was in line with previous home study [16] in that better satisfaction observed in lower income groups. Outside home, Malaysia, it was also positively linked as having no cost or low spending money for the ANC services had better services satisfaction [30]. The synonymous finding could probably indicate that low costly services are more satisfying to the poor ANC clients here and there.

Women in married marital status were significantly associated (AOR = 8.17, 95% CI; 1.43, 46.5) for having satisfied to Skilled Antenatal Care services. No other studies before could have observed this association directly. In this study, we could explain that this significant association could probably be related to existence of high number of married women among the study participants. This was because, married marital status and ANC service utilization had significant association in Ethiopia [31, 32].

This study revealed that having satisfied to Skilled Antenatal Care services were statistically significantly associated with being from Welayta (AOR = 0.26, 95% CI; 0.13, 0.54) and other (other includes: Amhara and Zayse) ethnic group (AOR = 0.2, 95% CI; 0.07, 0.61) respondents. This ethnic association with maternal Skilled Antenatal Care services satisfaction was also linked by Kenyan, Sri Lanka and Nigerian studies [33–35]. This particular ethnic discrimination could probably be related with non-native ethnic group’s perception as being discriminated for services quality. This was because there was no one from minor ethnic group reported for ethnic related discrimination in FGDs. Concerned bodies needed to make specific interventions to poorly satisfied ethnic groups and further study is required.

This study shows that respondents who were in the fourth ANC visit were significantly associated (AOR = 9.02, 95% CI; 1.76, 46.1) for good-satisfaction to Skilled Antenatal Care services. This was supported by finding of Riyadh [12]. High good-satisfaction with highest visit gives the women opportunity to ask her concerns and this could increase her good feeling towards the services. Besides this, high number visit could possibly enhance positive relationship between providers and client, making maximum good feelings or satisfaction towards the women. This probable explanations were also supported by Tanzanian finding [36]. As satisfaction can be one of the effect for quality, perceived good quality services was also associated to that of subsequent ANC visit by a woman, as one home study suggested [37].

### Strengths of the study

Being facility based is an advantage for better representing of the study district on the outcome variable as compared to being community based house to house survey. This was because it included respondents in the most recent service use. So, recall bias was highly minimized. Being triangulated design was also the strength. Moreover, being professional data collectors (nurse) used was an advantage for effective collection of obstetrics related information from the respondent’s as it is difficult for non-health professionals.

### Limitations of the study

Design related cause-effect relationship for all significant associations may not be established. Being facility based interviews could be disadvantageous in that it inhibits criticism of medical care by some of respondents even if it had weighted advantage over recall bias minimizations. The woman could depend on a single satisfying service for decisions of overall satisfaction, even though her self-report was the primary option for capturing customer satisfaction data. Social desirability bias could have affected the quality of data collected because study subjects might get difficulty to answer dissatisfaction in the presence of an interviewer. This bias was minimized via interviewing in a separate room by non-staff members’ enumerator without wearing gown. Being non-scale based satisfaction measurement data collection tool could be the disadvantage for better observation of concentrated area in a scale of satisfaction. Moreover, women who were on a first visit could not be able to judge quality of some components of services accurately. This bias was reduced by clarification of components of services and allowing her to observe some amenities back again during the interview. Selection of questions and indicators could also have lead to a skewed interpretation.

## Conclusion and recommendations

Women who reported having good-satisfaction to overall Skilled Antenatal Care services were highest. The most common specific component of ANC that had good-satisfaction by the respondents was maintaining “Privacy” during examination (81.7%). The most common health education topic during ANC services which was satisfied by the respondents was “Diet and nutrition” (82.2%). Absence of sonar test, no doctor, and long waiting time in the center were commonest causes of dissatisfaction by client report. Woman birth order, distance to the nearest health center, previous ANC visit, family monthly income, marital status, ethnicity, and current ANC visit type were independent predictors of satisfaction to Skilled Antenatal Care services. Providers must re-plan and improve their performances in the district for another highest satisfaction in the future. Especial attention must be given to those ethnics other than Gamo in the district regarding overall ANC services, since dissatisfaction was highest among them. Policy makers and other stake holders should give attention to increase ANC visit coverage in the country by developing strategies with the aim of maximum satisfaction to Skilled Antenatal Care services. Government officials should be engaged in development of middle income economy for women through women economy development strategy and intervention throughout the country. Early ANC visitors should also be given attention by providers to increase chance for return visit through maximizing client satisfaction in every component of services.

## Abbreviations

ANC: Antenatal Care
FGD: Focus Group Discussion
HE: Health Education
IEC: Information, Education and Communication
